# Bridging the research–practice gap in healthcare: a rapid review of research translation centres in England and Australia

**DOI:** 10.1186/s12961-020-00621-w

**Published:** 2020-10-09

**Authors:** Tracy Robinson, Cate Bailey, Heather Morris, Prue Burns, Angela Melder, Charlotte Croft, Dmitrios Spyridonidis, Halyo Bismantara, Helen Skouteris, Helena Teede

**Affiliations:** 1 Monash Centre for Health Research & Implementation, School of Public Health & Preventive Medicine, Monash University, Level 1, 43-51 Kanooka Grove, Clayton, Victoria, 3168 Australia; 2 School of Nursing, Midwifery & Indigenous Health, Charles Sturt University, Bathurst, NSW 2795 Australia; 3 Monash Partners Academic Health Science CENTre, Clayton, Victoria Australia; 4 School of Management, College of Business, RMIT University, Melbourne, Australia; 5 Monash Health, Clayton, Victoria Australia; 6 Warwick Business School, Gibbet Hill Road, Coventry, CV4 7AL United Kingdom

**Keywords:** Research Translation Centres, leadership, workforce development

## Abstract

**Background:**

Large-scale partnerships between universities and health services are widely seen as vehicles for bridging the evidence–practice gap and for accelerating the adoption of new evidence in healthcare. Recently, different versions of these partnerships – often called academic health science centres – have been established across the globe. Although they differ in structure and processes, all aim to improve the integration of research and education with health services. Collectively, these entities are often referred to as Research Translation Centres (RTCs) and both England and Australia have developed relatively new and funded examples of these collaborative centres.

**Methods:**

This paper presents findings from a rapid review of RTCs in Australia and England that aimed to identify their structures, leadership, workforce development and strategies for involving communities and service users. The review included published academic and grey literature with a customised search of the Google search engine and RTC websites.

**Results:**

RTCs are complex system-level interventions that will need to disrupt the current paradigms and silos inherent in healthcare, education and research in order to meet their aims. This will require vision, leadership, collaborations and shared learnings, alongside structures, processes and strategies to deliver impact in the face of complexity. The impact of RTCs in overcoming the deeply entrenched silos across organisations, disciplines and sectors needs to be captured at the systems, organisation and individual levels. This includes workforce capacity and public and patient involvement that are vital to understanding the evolution of RTCs. In addition, new models of leadership are needed to support the brokering and mobilisation of knowledge in complex organisations.

**Conclusions:**

The development and funding of RTCs represents one of the most significant shifts in the health research landscape and it is imperative that we continue to explore how we can progress the integration of research and healthcare and ensure research meets stakeholder needs and is translated via the collaborations supported by these organisations. Because RTCs are a recent addition to the healthcare landscape in Australia, it is instructive to review the processes and infrastructure needed to support their implementation and applied health research in England.

## Introduction

“*If you think competition is hard, you should try collaboration*” (Kings Fund Report, 2019)

Over the past decade, there has been wide international concern that new health research and evidence is not translated into practice in a timely fashion [1, 2]. The 17-year time lag between evidence and clinical practice change has been widely touted [3]. Systemic barriers such as lack of integration between health and research, dissonant metrics, organisational and professional silos, pervasive competition, lack of collaboration, and a failure to engage relevant stakeholders have all been identified as contributors to translation ‘gaps’ [4–6]. An international response to accelerate the translation and mobilisation of new knowledge has been the development of large-scale partnerships between universities, research institutes and health services that aim to integrate healthcare, research and education [7]. In world-leading United Kingdom and Australian health systems [8], these partnerships include a focus on evidence translation and health impact.

In England, these ‘partnerships’ include Collaboration for Leadership in Applied Health Research Centres (CLAHRCs), Academic Health Science Centres (AHSC) and Academic Health Science Networks (AHSNs). Collectively, these entities are often referred to as Research Translational Centres (RTCs) and they have been established internationally in the United States, Canada, England and Australia. In 2008, the National Institute for Health Research (NIHR) established nine CLAHRCs to increase the uptake of promising clinical research into practice and improve outcomes by engaging service users and the public in applied health research [9]. CLAHRCs competed with each other for NIHR funding in 5-year cycles. Subsequently, AHSCs were established in 2009. They are not formally part of the NIHR and, unlike CLAHRCs, did not receive NIHR funding [9]. These centres originally developed through interactions between rival institutions and occurred in a policy context that supported and accredited a limited number of prestigious AHSCs that continue to operate in strong institutional competition [10].

In 2013, a second round of competitive CLAHRC funding saw the recognition of 13 centres across England. Simultaneously, AHSNs were established with clear structures of accountability and budget and a focus on promoting and adopting innovation in healthcare. Commissioned by the National Health Service (NHS), concerns that the future of these networks may be constrained by budgetary pressures have been expressed [11], even though improving the uptake of innovation is valued in improving the quality and sustainability of healthcare in England. CLAHRCs were tasked with strengthening collaborations with the AHSNs [9]. A third round of CLAHRC funding, announced in 2019, saw the centres renamed as Applied Research Centres (ARCs), with increased focus on social care and public health. Strengthening the links between the ARCs and the AHSNs remains a priority, with AHSNs expected to take up and implement evidence generated by the ARCs.

In Australia, the McKeon review (2013) identified that the best performing health systems are those that embed research in healthcare and recommended the establishment of integrated RTCs that combine hospital networks, universities and medical research institutes [12]. The review also recommended a doubling of investment in medical research to grow applied health research that drives efficiency and impacts on communities. Since 2015, the National Health and Medical Research Centre (NHMRC) has accredited seven Advanced Health Research Translation Centres and three Centres for Innovation in Regional Health (CIRHs) to encourage leadership in health research and implementation [13]. The accreditation process is competitive to a benchmark but RTCs do not compete against each other. The Advanced Health Research and Translation Centres and CIRHs are, to some extent, modelled on RTCs elsewhere, including England, but are uniquely ‘health service-led’ collaborations. The CIRHs have a specific focus on the healthcare needs of regional and remote Australian populations.

Another unique feature of the RTCs in Australia is that they have developed a national alliance – the Australian Health Research Alliance (AHRA). The Australian Federal and State Governments have since invested in these RTCs across the AHRA. Funds are shared equally across all RTCs accredited by the NHMRC and, hence, the system enables collaboration for greater benefit from existing funding rather than promoting competition. The AHRA has increasingly prioritised research on RTC operations and activities, including how best to mobilise strategic prioritised health research in practice and how to measure and capture impact. This is because, despite significant government investment, the optimal collaboration models and activities are yet to be fully understood, especially in Australia where the RTCs and AHRA are relatively recent constructs. In England, several evaluations of the CLAHRCs and AHSCs have been undertaken [9, 14–16] but these have mostly been internal evaluations and limited in scope. Given that both England and Australia have world-leading universal health systems [8] and that the recently established Australian centres are modelled on the English centres, a rapid review of RTCs (confined to England and Australia) was conducted to inform the ongoing development of these partnerships.

This rapid review is timely, with the CLAHRCs and AHSNs in England focusing on greater collaboration and the Australian centres recently being funded $300 million over 10 years, with a clear need for more research to guide evolution. Knowledge ‘gaps’ identified by Australian RTCs include workforce development, strategies for consumer and community involvement (CCI), optimal collaborations, governance arrangements and structures to drive collaboration. CCI and workforce development needs are diverse, yet here we focus on strategies aligned with the RTCs’ aim to integrate research and healthcare and to build collaborations and drive evidence-based healthcare improvement.

## Methods

Rapid reviews have emerged as an efficient way of supporting health policy-making and systems development by providing evidence in a timely and cost-effective fashion [17]. They employ a wide variety of methods [18] and, although we acknowledge that rapid and limited evidence searches can lead to missed information, these methods were chosen as pragmatic and timely and because they capture both academic and grey literature. Traditional systematic review processes were not amenable to the time-frame required by our health partners (the AHRA) and would not capture the diverse reports and evaluations found largely in the grey literature, although it is acknowledged that the grey literature is not rigorously peer reviewed and that combining published and grey literature may lead to bias [19]. However, rapid reviews do meet the needs of end-users in addressing emerging issues within limited time-frames.

The scope of this review included the vision, governance and structure of RTCs, their CCI, (public and patient involvement (PPI) in England), and workforce development strategies. This review included published academic and grey literature with a customised search of the Google search engine and RTC websites. Since abstracts were unavailable for reports in the grey literature, executive summaries, recommendations and table of contents were reviewed. We searched for academic publications in EMBASE and SCOPUS databases using the following search terms: “Collaboration for Leadership in Applied Health Research and Care” OR “Academic Health Science Centre*” OR “Academic Health Science Network*” OR “Advanced Health Research and Translation Centre*” (acronyms were excluded, as they failed to yield results). In terms of the grey literature, the above terms linked to “AND evaluation” were searched on Google, then sorted by relevance. We also searched the websites of RTCs in England and Australia.

The search period was limited to the previous 10 years (2008 to August 2019) to ensure currency of our findings in a landscape where RTCs continue to evolve. Inclusion criteria for the published and grey literature included reports or evaluations that addressed structure, governance, community and consumer engagement, and/or workforce development. Although the heterogeneity of grey literature means it is less amenable to traditional forms of analysis, it did extend the scope of findings by incorporating information on the applied topic areas and by filling gaps that were apparent in the academic literature. Permission to conduct this study was received from the Monash University Human Research Ethics Committee.

## Results

**Fig. 1:**
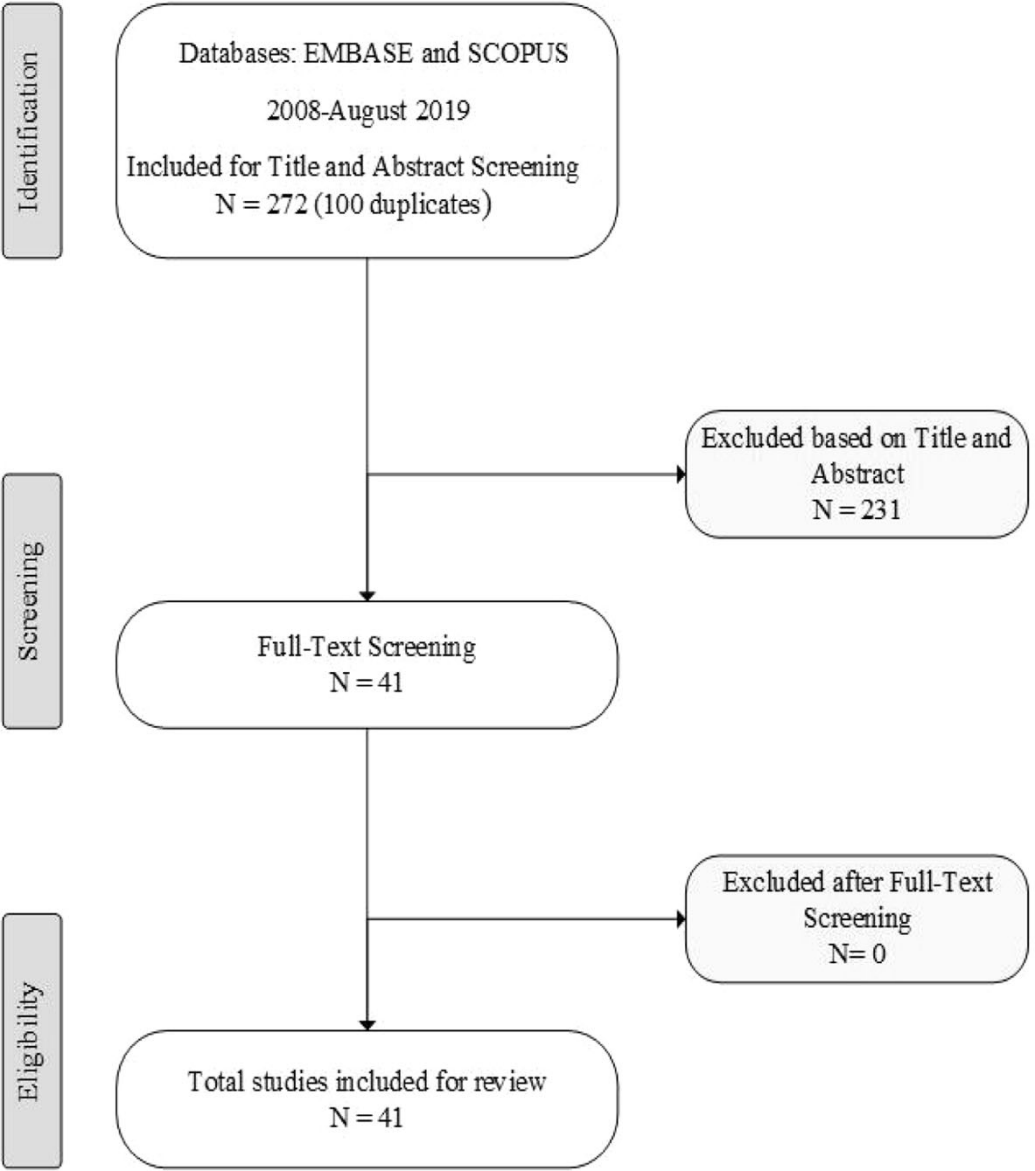
PRISMA diagram

A search of EMBASE and SCOPUS identified a total of 272 relevant papers (after duplicates removed) over 10 years (2008 to August 2019). A review of titles and abstracts identified 41 scientific papers for consideration, all of which addressed the evaluation domains of interest and were retained after full-text review, as shown in the PRISMA diagram in Fig. 1. This included one systematic review of CLAHRC evaluations [20] but no evaluations of RTCs in Australia. The evaluations of CLAHRCs were diverse, and often descriptive and exploratory in nature with a paucity of evidence about the overall impact of centres, particularly in relation to knowledge mobilisation processes [20]. Of the evaluations reviewed, most focused on partnerships, structures and processes. Likewise, a scoping review of AHSCs found most of the literature to be descriptive case studies or commentaries [7]. This highlights the challenges involved in evaluating complex systems that may require different methods (such as social network analysis) to better capture their dynamics [20]. The grey literature and review of all RTC websites provided additional information specific to each centre.

### RTCs’ vision, governance and structure

Although RTCs share a common aim to integrate research and training with health services, there was considerable variation in their vision, governance and structure in both countries. In England, the CLAHRCs have a declared mission to support high-quality applied research that meets the needs of local health and care systems [21], yet there was considerable variation across individual centres. Table 1 demonstrates that collaboration for patient benefit, translation and the harvesting of evidence were commonly identified in the vision statements for CLAHRCs, while the AHSNs had a focus on innovation as a key part of their mission. The AHSNs were created to connect the NHS and academic organisations, local authorities and industry with a clear focus on improving patient outcomes [22]; they aim to foster opportunities for industry to work effectively with the NHS by leading regional networks and generating economic growth in their regions. The AHSCs in England share a similar aim to improve health education and patient care and are commonly ‘nested’ within an AHSN but their focus is more on research excellence and the translation of new innovation from the laboratory to the bedside. Governance structures in England appear well developed, albeit highly variable. Most RTCs had all partners represented on their governing boards, with specific steering, advisory and PPI committees. The AHSNs reported over-arching executive boards with discrete advisory committees that help define and advise on regional issues and the inclusion of clinical commissioning groups in their governance. The governance and structure of AHSCs was variable – some reported having academic leaders who determined themes, while others reported having equal representation from all partners.

**Table 1:** Website search of centres: vision, structure and governance, community engagement and workforce capacity-building

Centre	Vision (Mission, purpose or statement)	Structure and governance	Community engagement (CCI/PPI)	Workforce capacity-building
CIRH 1	Website statement: “The … is a partnership between health services, health/medical research organisations and educational institutions.”	10 members, including research centres, universities and health services. Governed by a committee with a chair and membership of the founding partners and a director	Not stated	Not stated
CIRH 2	“Our Objective is to make appreciable improvements in patient outcomes and experience by translating evidence into practice” “Our Purpose is to accelerate the translation of evidence into practice to improve the health and wellbeing of regional, rural and remote communities.”	7 members, including research centres, universities and health services. Governing board (8 members), supported by advisory translation committee (7 members), which oversees the translation working groups and a director	Not stated	Not stated
AHRTC 1	“The purpose of [the centre] is to connect researchers, clinicians and community to innovate for better health.”	10 partners, including research centres, universities and health services. Governed by a council (strategy setting, 10 members), an executive (operational, clinical, academic and managerial committee of 18 members), an executive director	Consumer and community involvement (CCI) seen as vital. CCI personnel are actively involved in development of priorities, practices, policies, and research questions. Webpage devoted to CCI with videos and information	Centre delivers courses on good clinical practice (no charge), an implementation science masterclass, and a women in leadership programme
AHRTC 2	“The mission of the [centre] is to lead health translation through innovation.”	17 partners, including research centres, universities and health services Board (7 members including executive director); Council and executive director (21 members)	Not stated	Seminars offered for study co-ordinators, Audit training, plus training in conjunction with the local research platform
AHRTC 3	“Our vision is to transform the way research improves patient care and public health in our health system through strong collaboration, inclusive thinking and an overriding commitment to meet the health needs of our community.”	14 partners, including research centres, universities and health districts and hospital. The peak body is the governing council (12 members), executive director and management team	Not stated	Has conducted a symposium on Research Translation in a Complex Health System, and an Annual Forum
AHRTC 4	Website statement: “Working together to promote good health and wellbeing”	14 partners, including research institutes, universities and health districts and major teaching hospitals. Council of 22 members and director	Not stated	Aims to deliver top quality education and professional practice across partner organisations, no details stated
AHRTC 5	“Our vision is to be recognised as a premier academic health sciences partnership that is a global example of outstanding health services delivery.” “Our mission is to integrate innovative research with education, training and clinical care to deliver the highest quality healthcare for our local and extended communities.”	10 partners, including research institutes, universities and health services and hospital. Governed by a Board with representatives from each of the partners (10 members), plus Theme leaders (15 members) and an executive director	Not stated	Education stated as the cornerstone of an AHSC. Centre offers a full range of training and education relating to implementation science, research techniques and practical application. Activities are not specified on the website, but there is an education working group
AHRTC 6	Vision: “Continuously enhance the rate of translation of health and medical research into health care to create a self-improving, sustainable and high-quality health care system.”	10 partners, including research institutes, universities and local health networks and hospital. Detailed graphic of the overview of governance and operations on website, overseen by a board of partners (11 members)	Has specific webpages for CCI, describing how CCI is included in the work, a CCI framework, and a downloadable Community Engagement Report. There is a separate webpage for those interested in community engagement opportunities	Two streams: “Workforce development and training across Research Translation agenda” and “Staff development opportunities through fellowships, awards, and exchanges”, no specifics
AHRTC 7	“Our vision is to ensure health and medical research is continuously and rapidly translated into health care in order to create a sustainable, evidence-based, high quality health system.”	20 partners, including Universities; hospitals, research institutes, and other, plus 8 Associate Partners Governance: Executive Board (high level goal setting- 9 members), Management Committee (reps from each partner organisation) and Executive director	Offers courses on CCI. References to a separate website of the Consumer and Community Health Research Network	Education and training steering committee oversees offerings of an expanding suite of online training programmes, offered free to partner organisations
CLAHRC 1	“The mission of the …CLAHRC…is to work collaboratively with Partner organisations and other stakeholders including members of the public to co-produce and conduct high-quality, leadership enhancing, applied research designed to decrease health inequalities and improve the health of the population [in this area].”	21 partners, including Universities; hospitals, research institutes, and other. Steering Board (responsible for strategic direction and governance, 26 members), plus a three person external advisory committee	There are several webpages dedicated to public involvement in research, a link from front page titled “Get involved”, a video on public engagement, and a PPI newsletter	A free workshop on Effective Public & Community Involvement in Research is delivered, plus evaluation workshops and a Partner Priority bringing frontline professionals, researchers and leaders from Partner organisations together
CLAHRC 2	Website statement: ”Improving the health and wealth of [area] and the nation through research.”	14 partners, including commissioning groups, county councils, support group sand other, plus 7 University affiliated groups. CLAHRC director and admin: (8 members), Theme leads (6 members), Management Board (4 members), Patient and Public Involvement (PPI) (3 members)	PPI is emphasised: page of info and links for researchers on how to involve the public in research, a PPI newsletter, and a downloadable PPI strategy document	Webpage for training aiming to develop skills for “health, public health and commissioning workforce, and patients and members of the public” through courses and funding of fellowships
CLAHRC 3	Mission “To create lasting and effective collaborations across health and social care organisations, universities and local authorities to improve the services we can deliver for patient benefit.”	17 current partners, including NHS Foundation trusts, city and county councils, and Universities, 5 new partners, and four in negotiation. Detailed management structure graphic on website, and includes the following: theme steering committee, programme steering committee, programme executive committee, scientific advisory group and PPI supervisory committee	Extensive information about Patient and Public Involvement and Engagement (PPIE) on three main areas: Involvement, engagement, and participation, and includes PPi recourses and a latest opportunities page	Capacity-building webpage includes fellowships, PhD scholarships, and a MSC in Health Research Methods
CLAHRC 4	Website statement: “Investigating the best way to make tried and tested treatments and services routinely available.”	8 partners, including 2 universities, four NHS foundation trusts and 2 other. Detailed downloadable diagram on CLAHRC structure available. Governed by an Executive (23 members) which is accountable to the Board (21 members) Public and Patient Involvement Strategic Oversight Group (PPI SOG) is part of governance structure (23 members) External advisory board reviews projects (11 members) Executive Director	Section of website with a number of webpages for PPI, on involving patients, service users and families. Includes publications and PPI research projects	Training junior researchers appears to be a main aim. The CLAHRC offers an Implementation science masterclass and in 2018 organised an Implementation Science Research Conference, to be run again in 2019. The CLAHRC offers short courses for health professionals and financial support for research students
CLAHRC 5	Website statements: “Bridging the gap between research and frontline care” “Health research today improves lives tomorrow” “Harvesting evidence to ensure best practice in healthcare”	56 partners, including 19 National Health Service, 3 community trusts, 8 acute trusts (hospitals), 5 mental health trusts, 1 ambulance trust, 7 industry partners, 7 city councils, 9 universities, and 6 other. Universities included in this list are also associated with other CLAHRCs. There appear to be 2 main universities involved. Governance information not apparent	Brief webpage on Public Involvement with downloadable Public Involvement Strategy	No evidence of workforce capacity-building
CLAHRC 6	Website statement: “We are a collaboration of academics, clinicians and managers who undertake high quality applied health research focused on the needs of patients and service users, supporting the translation of research evidence into practice in the NHS and social care.”	Only academic partners are featured on the website (3 universities) Diagram on webpage detailing the governance structure, with a Board (7 members) and management executive group, executive committee, scientific advisory board and director	Webpage on community engagement with general information	One webpage on research capacity development. The CLAHRC awards fellowships, runs events on applying for funding
AHSN 1	Vision: “Igniting innovation - bringing together the trusts, universities, industry, third sector and social care to transform the regional health and stimulate economic growth.”	Encompass 87 health institutions and nine universities. Hosted by a university hospital trust. The central management consists of the board (5 members) and executive personnel (> 22 members), distributed in senior, innovation, patient and public engagement and, patient safety collaborative, and communication team. Steering and operational management groups in each project which are responsible to the board	Website accommodates a specific page for patient and public involvement (PPI), informing various engagement models for co-production. Establishing a PPI senate. Patient and public-related events and newsletters	Theme-based training activities, specifically on enhancing junior doctor and pharmacist’s prescription quality
AHSN 2	Website statement: “Promote health service innovation and improvement by spreading innovation, improving health, and generating economic growth.”	Formed by six universities, 13 clinical commissioning groups (CCGs), 24 trusts, 11 local authorities, five enterprises, three life science sector support partners, two trade partners, and six other organisations. Overseen by the board (6 members) and operated by seven executives, i.e. CEO, COO, CCO, and directors on commercial, communications, patient safety, and health informatics. Advisory groups provide suggestions to the board in defining regional challenges and their solution	Facilitation of citizen’s senate as public representatives, which the network provides leadership training. Public participatory programme to gather opinions on activity ideas and issues	Priority-based training and development, utilising workshops and website-based approaches
AHSN 3	Website statement: “To be a recognized international leader in accelerating innovation to improve citizens’ health and wellbeing.” Objectives: Continuous health innovations, advancement of healthcare delivery, academic and industry engagement, influence policies	Consist of CCGs, trusts, universities, research centres and network, industries, national bodies and investment agencies. 18 board members, with six executive teams (CEO, CAO, digital innovation officer, management director, academic director, and clinical director). Operational activities conducted by senior management team (13 members)	Specific page on public involvement and engagement on website. Establishment of Patient experience group (PEG) which involved in co-producing ideas and activities. Website does not provide methods to register as public and patient contributors	Various trainings on translational research and its programme management, health data science, and genomics. Massive Open Online Course (MOOC) on Clinical Bioinformatics
AHSN 4	Vision: Building a health innovation-driven future by connecting, inspiring, and supporting people with great ideas. “Everyone benefits from the best in health and care.”	Developed by 55 organisations, including trusts, CCGs, higher education institutions, industries, authorities, patient and charity organisations, and other third sectors. The board (19 members) navigate the executive teams, consisting of a chairman, accountable officer, chief officer, commercial director, and medical director). Operationalised by eight senior leaders	Not stated	A webpage dedicated to capacity-building, informing focused human resources training, leadership programmes, courses, and fellow opportunities. Six Community of practices (CoP) developed by engaging 18 trusts and 14 organisations
AHSN 5	Website statements: “Turn the potential of innovation into reality to help solve pressing challenges by collaborating across the health sector” “Accelerate the adoption and spread of innovation amongst our member organisations and beyond.”	Formed by three universities, nine trusts, and eight CCGs. Directed by the board (14 personnel) and managed by 31 executives. Specific committee on value creation, consisting of 6 members from industry and international health experts, to assist board decisions	Not stated	Intrapeneur programme on European healthcare and acute care. Courses and masterclasses in health innovation, value-based quality improvements, population health, and integrated care
AHSN 6	Vision: “Spread healthcare innovation faster within the regions.” “Bringing together organisations and individuals to save lives, increase the number of people getting the best healthcare, and contribute to a vibrant local economy.”	NHS and independent health providers, 21 CCGs, nine universities, six local authorities, and industries as its members. The chair and board are responsible to the members, and supported by committees. Managing director is accountable to the board, with support from executive team and delivery boards. Clinical leads become front-line in the network’s activities, and managed by the director. Members obey to financial delegation scheme in supporting the board’s operations	Community engagement plan for research development. No definite actions for community engagement informed in the website	Embedded in prioritised programmes, such as coaching for sustainability and transformation on health planning
AHSN 7	Website statement: “Assisting members to identify, evaluate, adopt, and disseminate transformative innovation.” “Assisting industries to gain expertise in developing, testing, and deploying products and services.”	Formed by trusts, CCGs, universities, and industries. The Board includes seniors from NHS, Clinical Commissioning Group (CCGs) and Trust employees from across the NENC region. An executive team (4 members), its supporting core team (38 members), and health improvement leaders working under the boards. Executive team manages networks of practitioners	Not stated	Collaborative learning on priorities issues, such as maternity and neonatal and deteriorating patient. Innovation showcase programme for knowledge dissemination
AHSN 8	Website statement: “Working together for patients; respect and dignity; commitment to quality of care; compassion; improving lives; everyone counts.”	22 providers, 20 CCGs, nine universities industries and business partners. Board of partners, as “ambassadors for innovation”, are formed to plan and monitor the network. Activities managed by three non-executive directors, six executive directors, and 46 supporting staffs	The network provides specific personnel on public involvement. Formation of public involvement and engagement senate, which has power to influence patient safety, innovation testing, and technology-based development. Website accommodates public registration for senate position, information on events and surveys	Coaching academy: Capacity-building programme by workshops and online learning. Webinar-based discussions
AHSN 9	Website statement: “Improving health and generating economic growth.” “Meeting local health needs through the spread and adoption of innovation.”	Hosted by university hospital trust. Consists of five CCGs, ten providers, eight collaborative organisations, four universities, seven research institutes, industries, enterprises, and patient organisations. Governed by the board and managed by an independent chairman. Other personnel include CEO, COO, and oversight group chairs	Facilitation of an oversight group for patient and public involvement, engagement and experience (PPIEE), responsible in forming patient experience group. Leading together programme, engaging public, patient, and staff to discuss potential network activities. Patient and public involvement training programme	Trainings included in patient safety, clinical improvement, and clinical innovation programmes
AHSN 10	Website statement: “Improve the health and patient experience of people in the region by supporting and accelerating innovation and quality improvement”	17 full members and 10 associate members. Board of directors (11 personnel) appointed from full members. Associate members included in generating ideas disseminating the activities. Operational activities conducted by staffs from academia, front-line care, research, IT, analytics and support services	Website-based open recruitment for public and patient involvements. Establishment of quality improvement partner panels launched in July 2018	“Spread academy”: Training health professionals to reform health in a large-scale. Webinar-based training related to innovation generation
AHSN 11	Website statement: “Bring greater improvements to the entire healthcare pathway.” “Supporting the system to implement and evaluate integrated pathways and new models of care.” “Patient-centred approach.” “Develop capability and infrastructure to improve quality, patient safety and experience.”	40 organisations across the trusts, higher education, local authorities, patient groups, CCGs, the third sector, government and industries. The board led by a chair and managing director, with inclusion of senior representatives across the partnership. The board direct executive group (30 personnel), audit and risk committee, nominations committee, and remuneration committee	Systematic efforts on partnering with marginalised communities, including capacity-building, information provision, and impact evaluation. Engaging public into “Journal club” to discuss academic literature produced by the network. Facilitating online platform for patient feedback. Public inclusion to the boards, committees, groups or projects, including steering groups in the involvement	Capacity-building programmes in end of life care, genomics, and healthcare quality
AHSN 12	Website statement: Improve health, achieve excellence, and boost innovations. “Connect academics, trusts, industry and others to bring fresh energy to old problems, inspired thinking to new ones and to spread innovation and best practice.”	Built by 11 trusts, four universities, 10 CCGs, and 8 local stakeholders. The board (14 members), involving all partners, provide direction and oversight of the work. 12 senior staffs drive activities, leading the team of academia, frontline care providers, researchers, analysts, and support services. The staffs are accountable to the board	Systematic public and patient involvement for co-production and co-design of programmes by (ARISE+ model). Patient experience library as the source of information for policy-makers. Establishment of public panel for quality improvements. Direct patient engagement activities	Theme-based training in psychiatry for junior doctors and capacity- building for primary care workers
AHSN 13	Mission statement: “Lead, catalyse and drive co-operation, collaboration and productivity between academia, industry, health and care providers and commissioners, and citizens.” “Accelerate the adoption of innovation to generate continuous improvement in the region’s health and wealth.”	Two type of memberships: Free and paid, with differences in the benefits, services, and premium access. Performed by the board (13 members) and executive team (11 members). No explanation on detailed governance in the website	Public engagement is defined in the network’s statement. No explanation on approaches to involve the public and patients	No specific explanation related to capacity-building. Online platform facilitates sharing of health innovations across members
AHSN 14	Website statements: “Driving the development and adoption of new innovations.” “Enabling patients to play an increasing role in their own care and of others.” “Impact-oriented partnerships.”	Formed by 14 trusts, five CCGs, three universities, local partnership, and integrated care system. The board (15 members) navigates the network, with an academic as the chair. Operations are managed by senior team (10 members)	Co-created coordinated approaches for public involvement within the region. Public representatives in the board. Five public members as advisors. Activity-based engagement, as in toolkit productions and consultations	Website-based approach by toolkit development for clinical decision making, communication strategies, and quality improvement
AHSN 15	Vision: “Improve the health and prosperity of our region by unlocking the potential of new ideas”	Managed by the team with 44 personnel. Led by a chair, supported by directors, programme leads, public and patient engagement lead, marketing and communication, managers, clinical advisor, analyst. Detailed governance not provided in the website	No details on methods to engage patient and public participation. One programme to accommodate patients’ voice found in September 2019	Establishment of an academy to provide training and resource access, focus on improving quality and patient safety
AHSC 1	Website statement: “Ensure patients reap the benefits of the world class research, clinicians and industry which are based on the region and surrounding area”	One university and three trusts as members. Five workstreams: Education, campus integration, research, philanthropy. Board decides and drives the vision and strategies. Executive group provides oversight in the implementation of the strategies. The group provide reports to the Board. Management office is accountable for managing and coordinating the activities, including finance, corporate governance, communication, office management, project, and events. Directed by the executive group	Not stated	Provision of training courses and tuition for members. Online learning platform for patients, carers and professionals. Establishing a surgical training centre for advancing hands-on experiences
AHSC 2	Website statement: “Accelerating the translation of basic science discoveries into patient and population health benefit.” “Deliver (inter-) nationally leading infrastructure and programmes in health research, education and clinical care.”	One university and three trusts. Joint governance from all partners and performed by the directorate. The Strategic Partnership Board (nine members: Three from university and two from each trust) is responsible for progress monitoring. Formed by board-level representatives. The Joint Executive Group, led by a director, is accounted for implementing strategies and managing the performance. Governance diagram provided in the website	Not stated	By various activities, such as seminar series, clinical academic training hub, and leadership development programme
AHSC 3	Website statement: “World-class research, education and clinical practice are brought together for the benefit of patients.” “Translate cutting-edge research and existing best practice into excellent patient care.”	Three trusts and one university. A university-led joint board is assembled to plan and drive strategies. Includes all trusts’ CEOs and four external non-executive directors. The board direct chief executive action group to develop strategies. Operational executives create activities from action group’s strategies. Programme office manages and coordinates the activities. Detailed structure provided in the website	Patient and public inclusion in defining outcomes	Capacity-building scholarship. Website-based learning hub. Interprofessional learning on patient safety
AHSC 4	Website statement: “Uniting leading healthcare providers with world-class academics and researchers.”	One university and five trusts. No access for governance details	Unable to access the main website	Unable to access the main website
AHSC 5	Website statement: “Create an environment where the best research can be immediately translated, applied and evaluated for patient benefit.” “Coordinate clinical and academic excellence within the partners.”	Two universities and two trusts. Five board members look after the theme delivery and high-quality research, care and education integration. Seven theme leaders and two senior management become the operators	No details on patient and public inclusion	Capacity-building by courses and CPDs. Joint training for digital developments, innovation and interprofessional training and development
AHSC 6	Website statement: “Combines the expertise to focus on selected specialist programs.” “Diffusion of innovation and best practice across our region.”	Formed by three universities and five trusts. The centre develops theme-based academic medical centres (AMCs) to support the implementation, formed by specialist hospitals and postgraduate institutes	Not stated	Not stated

*AHRTC* Advanced Health Research and Translation Centres, *CIRH* Centres for Innovation in Regional Health, *AHSN* Academic Health Science Network, *AHSC* Academic Health Science Centre

The stated vision of RTCs in Australia emphasised the integration of research with healthcare and partnerships. The translation of evidence was a strong and consistent focus, largely funded by the Medical Research Future Fund (MRFF) that provides grants for rapid applied research translation [23]. Early funding priorities have been identified by the MRFF and include reducing unwarranted variation, improving clinical pathways, improving the health of vulnerable groups, increasing primary care research and reducing risk factors for chronic diseases [23]. In terms of their structure and governance, RTCs in Australia appeared to have more consistency, with all partners represented on boards or councils and various advisory, translation or management committees. Healthcare leadership (rather than academic) was a key feature of Australian RTCs as a means of enhancing the accountability, relevance and impact of research. This governance structure is challenged by the fact that universities are federally funded, whereas healthcare is funded by state governments [24]. However, this is being addressed by the fact that both the RTCs and the AHRA are federally funded. One RTC in Australia has a unique ‘bottom up’ structure, where governance is strongly led by Aboriginal community controlled organisations and Aboriginal ‘voice’ is embedded across all levels of the organisation (the Central Australian Academic Health Science Network). Few RTCs in either country report on their websites how their vision or governance was developed or whether a strategic plan was in place.

In terms of structure, or the ‘architecture’, some RTCs were built around clinical themes (largely disease focused with flagship programmes), with some being structured around platforms or fields of work such as public health and health services. In England, leading figures with particular research experience acted as Directors and many centres reported having a three-tier structure with a Board, management committee and working groups that align with the clinical themes/projects. While RTCs in both countries identified diverse clinical themes, few reported information on how they developed priorities for themes or whether they involved collaborations with services users and healthcare providers to inform structures and processes.

### 3.2. Workforce development

The review identified that workforce capacity is being developed across the system, organisation and individual levels to build capacity in translational research and healthcare improvement. This requires leaders with broader skills and support to operate across organisational boundaries and address system-level barriers to change. In England, national efforts to develop leadership include the NHS Leadership Academy and NHS Horizons, which collaborate to identify future leadership development directions [25]. While the Horizons team supports leaders of change, the Leadership Academy provides a range of tools, models and programmes to support individuals and organisations to develop leaders [26]. In Australia, there is no coordinated national effort but some initiatives are emerging. In this context, Table 1 demonstrates that RTCs in both countries are all undertaking workforce capacity-building. At the individual level, diverse training needs were identified, including research and data skills, CCI and translation literacy.

The literature confirms the focus on and importance of skills in implementation research, knowledge mobilisation, evaluation skills and collaborative priority-setting with potential end-users of research [3, 27]. Time and space are needed to build effective collaborations and, while the ARC model did facilitate collaborative priority-setting, Cooke et al. [27] reported that scant knowledge exists about processes or guidance on how best to achieve meaningful collaboration. Platforms for negotiation and decision-making (such as special interest groups and advisory groups) were possible enabling factors, as were formal consensus methods for priority-setting [27]. In England, the James Lind Alliance brings patients, carers and clinicians together to identify research priorities [28]. In Australia, Delphi and Nominal Group Techniques have been adapted and used for eliciting priorities across stakeholders [29, 30].

In England, an important organisational workforce enabler for meaningful engagement, embedding research into healthcare and the translation of new evidence, was leadership. Leadership was identified as a key factor in the overall success of RTCs, including in their workforce capacity for knowledge mobilisation [20, 31–33]. Currie et al. [33] stressed the importance of understanding the social position of senior members of CLAHRCs. Although well-known clinical academics are likely to lead the centres, this study found that privileging pre-existing relationships may constrain much-needed change and meaningful engagement with service users and frontline clinicians [33]. Leadership in CLAHRCs has been enacted in three ways: ‘push’ models for top down leadership that focus on technical infrastructure, pull methods that aim to increase leadership capacity among project leads and more collective approaches that dispersed leadership to drive new relations between academia and clinical practice [32]. Aligned with this, a recent Kings Fund report highlights the importance of system leadership (being comfortable with chaos) in driving meaningful change [6].

Although dispersed leadership approaches were crucial for the exchange of new knowledge, push and pull models continued to influence how knowledge was ‘moved’ within CLAHRCs, especially in relation to the development of technical infrastructures and translating knowledge at the project level [32]. While more distributed models of leadership were associated with increased potential for engagement with the CLAHRCs [20], a longitudinal realist evaluation of three centres found that a blend and alignment of designated leadership with distributed leadership was a necessary condition for collective action and implementation [34]. The presence of both these leadership styles appeared to be important for ensuring alignment and integration across streams [34]. As such, workforce development in leadership appears important in the context of RTCs.

The need to move knowledge across professional ‘silos’ resulted in several RTCs creating new system approaches such as knowledge-brokering roles (although they varied considerably across centres) [20, 35–38]. For example, some deployed ‘diffusion fellows’, who were senior health staff seconded to actively bridge the research–practice gap [35]. Despite showing much promise, knowledge brokering and other hybrid roles were often unrecognised and lacked support within their organisations [39, 40]. Although management theory identifies that knowledge mobilisation relies on relationships and is an inherently social undertaking [9, 41], the deployment of hybrid roles as a means of overcoming system barriers requires particular capabilities and was found to be challenging [20]. Nevertheless, workforce capabilities, such as stakeholder engagement, co-design, collaboration and team-work, and the co-production of knowledge, rely on understanding complexity and working across multiple levels (individual and organisational) to enact new knowledge [42]. The importance of developing skills for mobilising knowledge across disciplines and different users was confirmed in the literature [27, 33, 43–47]. Mobilising knowledge that is multidisciplinary requires different communities to interact [15] and RTCs are well placed to enable this kind of cross-silo collaboration, including with health, business, IT, social sciences, engineering and other disciplines.

Individual workforce capabilities for supporting RTC endeavours are not all technical and may include observational skills, appreciative inquiry, systems thinking, improved understanding of data, distributive or collective leadership, and quality improvement – all of which are increasingly found in English workforce programmes but are not yet incorporated into workforce programmes in Australia. At the level of the system and organisation, key workforce development approaches identified in this review include leadership and mentoring [48], processes for stakeholder engagement [27], and the creation of new hybrid roles to move knowledge across discipline and organisational boundaries. Despite a focus on leadership, the evaluation of three CLAHRCs by Rycroft-Malone et al. [49] identified that, on balance, they tended to conduct research rather than focus on ‘how’ to use and apply new research evidence. This means that closing the knowledge–practice gap and methods for translating evidence into improved patient outcomes are yet to be clearly established [49]. However, AHSNs are now more aligned with the CLAHRCs to increase the translation of generated evidence.

### CCI (Australia) and PPI (England)

One significant difference between Australian and English centres was the latter’s strong focus on PPI. England has a national PPI strategy, with PPI a policy and funding requirement and a key strategy for situating patients at the centre of research and healthcare improvement [27]. The importance of PPI in healthcare has been acknowledged for some time in England; however, there is still limited research on the optimal methods for driving and enabling PPI [34, 50]. The literature highlights a significant gap in understanding how PPI can inform implementation research that often focuses on the behaviour of health professionals and health systems and policies (as opposed to clinical research) [34]. Despite significant advancement in England, cultural barriers persist, including the narrowness of PPI models that fail to address empowerment, equality or diversity strategies [51]. Often, the level of PPI operates more as consultation rather than as active co-production and empowerment.

Other processes for authentic PPI enshrined in all CLAHRCs include providing payment for PPI representatives to attend meetings and training to enable more informed and active participation. The provision of training and remuneration for PPI representatives is a significant difference between England and Australia; however, real progress in PPI in England cannot be realised without an effective mechanism for coordinating efforts across the complex network of organisations that comprise the NIHR [51]. The systematic review conducted by Kislov et al. [20] reported that none of the NIHR-funded evaluations had a particular focus on PPI, although one included interviews with PPI representatives [9] and three investigated how PPI was enacted [52–54]. These evaluations all acknowledged the difficulties of quantifying PPI elements and Marston and Renedo [52] recommend the inclusion of patient voices and tracking dynamic social processes and networks to better understand the key elements and impact of PPI. It is important to identify the dynamic processes and networks through which PPI can contribute to healthcare improvement efforts [20] as well as the key time-points and strategies for PPI to have the most impact in the translational research cycle [51].

In Australia, only three RTCs included dedicated information on CCI on their websites. However, across all RTCs, the AHRA have prioritised CCI as a national system-level initiative and have developed a CCI strategy with key stakeholders and completed both an environmental scan of the literature and a national survey on the extent and nature of CCI. In 2018, a national workshop was convened to prioritise the next steps and RTCs committed funding and staff to collaboratively progress this work. To date, findings from Australia confirm that CCI is complex (consistent with the English experience) and that the locus of control for involvement in Australia remains largely with researchers [55]. The AHRA report also identified a need for more resourcing and better policy aligned with England. They recommended a range of strategies to promote and explore the value and impact of CCI. This report included the development of minimum standards for good practice in CCI involvement in RTCs and guidance on how to incorporate it across the research life cycle [55], alongside training and capacity-building. Currently, the report recommendations are being implemented collaboratively and co-ordinated nationally through the AHRA.

## Discussion

This review explored the visions, structures and governance processes of RTCs, their workforce development activities and CCI/PPI as key factors for integrating research with health service and community needs. Centres in both England and Australia share a common architecture in that they generally have boards that represent all partners and are organised along research themes that reflect their research strengths, with cross-cutting platforms to enable collaboration with health services. In terms of their vision, RTCs in England appear to have a greater research focus on innovation (AHSNs), collaborative and applied research (CLAHRCs), and a traditional push model of discovery and clinical research into practice (AHSCs). In Australia, RTC visions are aligned with translation, partnerships, and impact and have a strong and consistent focus on research translation.

In terms of workforce development (aligned with RTC visions to integrate research into healthcare, build collaboration and drive evidence-based healthcare improvement), leadership was a key enabling factor. Given that they are an amalgam of stakeholders with potentially competing demands, it is perhaps not surprising that leadership is a prominent theme. Leadership approaches appear to require both dispersed and distributed or top-down and bottom-up approaches to facilitate working collectively with multiple stakeholders [32, 36]. Collective and distributed leadership approaches have also been shown to enable healthcare improvement and transformational change [32, 56, 57]. Evaluation reports and published literature identified knowledge mobilisation as another key workforce skill for evidence translation. Historically, the evidence translation gap was perceived as a practice/service responsibility and challenge, rather than a problem of implementation or knowledge creation [34]. This highlights the need for systems approaches with a more nuanced understanding of how knowledge moves and can be brokered within complex organisations to enable improvement.

In England, structural solutions, such as the creation of new hybrid roles, has proved challenging – particularly in relation to working across all levels of complex organisations and diverse contexts [58–60]. However, skills and capabilities for moving knowledge in healthcare organisations were identified, including process and systems thinking, the involvement of stakeholders, change management, facilitation, negotiation, and advocacy skills [34, 61]. These are yet to find their way into traditional healthcare innovation and knowledge mobilisation roles, where the focus is often organisational and inward looking rather than collaborative with stakeholders and engaging with external evidence [40]. At this stage, workforce capacity development is more developed in English centres compared with Australia. However, Australian RTCs are now working together with nationally coordinated efforts to improve and scale workforce development activity.

Likewise, in England, PPI is well established and embedded in policy and funding requirements, although there is also a recognition that optimal processes for PPI and their impact should be better understood [14–16]. When utilised effectively, PPI appears to have the potential to transform services and address the research–practice divide [62, 63], but it is important to research and translate how patient input can be best integrated at all levels within and between RTCs. In England, funding, dedicated staff and training are available for both PPI members and frontline staff with co-design and co-production with stakeholders; this is not yet mirrored in Australia, where training programmes for the public and service users are emerging but remain under-developed. However, the AHRA has strongly prioritised and developed a national framework and is focusing on a coordinated approach to CCI. Funding bodies encourage but do not require CCI. One RTC in Australia, with community controlled Aboriginal health service members, appears to be leading in terms of processes for community engagement and clinical and corporate governance participation. Further research and evaluation are needed on the optimal methods and impact of CCI in research and healthcare improvement.

Overall, the findings from this review are important for the evolving RTCs in Australia, which are relatively young organisations and are due for re-accreditation by the NHMRC in 2022. Although this review focused on the structures, leadership, workforce development and engagement with communities of RTCs, it is important to acknowledge that these highly complex interventions, with their relational interactions and processes for collaboration, are often poorly captured and articulated in the literature. In order to understand these nuances, qualitative research is warranted as a means of capturing the range of activities and outcomes generated by these collaborative platforms. Australia has yet to evaluate their RTCs but it is notable that the Australian government has recently committed a 10-year funding strategy, which validates the perceived potential and importance of these entities and provides for long-term strategic planning. It also mandates more evidence-based approaches and the need for evaluation. The Australian MRFF was announced as part of the 2014–2015 federal budget and will build to a $20 billion perpetual fund over the next decade [64]. The MRFF scheme will complement and enhance current research funding schemes but will focus on delivering a health system fully informed by research with community and patient impact [65]. This approach is supportive of RTC visions and directly aligns with strategic prioritised research rather than conventional investigator-led research [66]. This is important because the systematic review of CLAHRC evaluations identified that 5-year funding cycles in England were insufficient to foster and embed collaborations between academic and service providers [20].

In Australia, the AHRA has prioritised streamlining and the consistency of structures and processes, whilst respecting regional differences. This Australian collaboration is possible in the context of avoiding direct competition for accreditation or funding. This has enabled a more collaborative approach to challenges and coordinated activities nationally within and between centres. This is consistent with recommendations from England that more research is needed that focuses on how collaboration occurs between RTCs [16] and with the recent Kings Fund report [6] on the vital need for more collaboration and less competition in healthcare improvement.

RTCs are complex system-level interventions that will need to disrupt the current paradigms and silos inherent in healthcare, education and research in order to meet their aims. This is likely to require vision, leadership, collaborations and shared learnings, alongside structures, processes and strategies to deliver impact in the face of complexity. The impact of RTCs in overcoming the deeply entrenched silos across organisations, disciplines and sectors needs to be captured at the systems, organisation and individual levels. Collectively, the creation of structures and streamlined processes to accelerate stakeholder engagement and collaboration, evidence synthesis, knowledge transfer, data systems and the effective integration of implementation and improvement into healthcare are the holy grail of RTCs. However, many centres appear to still focus on clinical themes and siloed projects. As these RTCs mature, capturing and learning effective ways to promote system change will rely on capturing higher level learnings from the plethora of RTC projects.

This includes better understanding of how to strategically prioritise research and how to build the capacity of the workforce to translate new knowledge into action. Recently, RTCs have developed novel ways of demonstrating these processes, including the use of ‘casebooks’ that detail the impact of research on NHS practice [67]. A consistency of purpose and activity is needed, alongside a focus on regional needs. Associated policy intentions and funding objectives that support shared learnings and collaborations are also important. Regardless of how RTCs are structured or where they are situated, these collaborative entities all share common potentials and challenges, mostly around how to collaborate in a siloed and competitive system and how to ensure that research and service delivery are integrated and evidence generated and translated for the benefits of the community they serve.

### Limitations

This rapid review synthesises diverse literature about broad and complex collaborative RTCs that have become key entities in policy and healthcare service improvement. Combining diverse information sources is challenging and, in the current review, may have limited the depth of findings. Although rapid reviews allow for the inclusion of grey literature, it is important to acknowledge that optimal methods for conducting these reviews are evolving and are yet to be determined. These reviews may lack rigour even while they may prove more viable in terms of cost, timeliness and the breadth of information accessed. However, there is a growing recognition that an understanding of systems perspectives and their inherent complexity require reviews from diverse sources and are not always well served by traditional approaches such as those afforded by systematic reviews [68]. The review only focuses on England and Australia as world leading universal health systems with strong policy and funding commitment to the integration of research and healthcare, evidence-based improvement and RTCs.

## Conclusions

A challenge for all RTCs is how to integrate research and healthcare and overcome competition to build collaboration and deliver impact. The English experience highlights that this requires a better understanding of the structure and vision of centres, their workforce capacity needs, and the nature of their collaborations with service users and communities. Although workforce capacity-building and the involvement of consumers and the community are more developed in England, the development of an alliance between centres in Australia is providing a platform for national coordination, shared learning and rapid collaborations. This alliance has facilitated and shared a national agenda in a range of areas. Given that the development and funding of RTCs represents one of the most significant shifts in the health research landscape, it is imperative that we continue to explore how we can progress the integration of research and healthcare and ensure that research meets stakeholder needs and is translated via the collaborations supported by these organisations.

## Data Availability

The datasets used and/or analysed during the current study are available from the corresponding author on reasonable request.

## Abbreviations

AHSC: Academic Health Science Centres
AHSN: Academic Health Science Networks
ARC: Applied Research Centre NIHR National Institute for Health Research
AHRA: Australian Health Research Alliance
CCI: consumer and community involvement
CLAHRC: Collaborations for Leadership in Applied Health Research
MRFF: Medical Research Future Fund
NHMRC: National Health and Medical Research Council
NHS: National Health Service
PPI: public and patient involvement
RTC: Research Translation Centres
